# Infrarenal Vena Cava Leiomyosarcoma Treated With Surgical Resection and Vascular Reconstruction

**DOI:** 10.7759/cureus.15808

**Published:** 2021-06-21

**Authors:** Yosra Malki, Hatim Lazaar, Tariq Bouhout, Badr Serji, Adnan Benzirar, Tijani El Harroudi

**Affiliations:** 1 Surgical Oncology, Mohammed VI University Hospital, Regional Oncology Center, Oujda, MAR; 2 Vascular Surgery, Medical School University Oujda, Oujda, MAR

**Keywords:** leiomyosarcoma, infrarenal vena cava, surgical resection, vena cava reconstruction, dacron graft

## Abstract

Leiomyosarcoma of the inferior vena cava is a rare malignant tumor with a poor prognosis. We report a case of a 39-year-old woman admitted for a surgical resection of a retroperitoneal mass revealed by pain localised in the right lumbar fossa. Computed tomography of the abdomen revealed a heterogeneous retroperitoneal mass compressing the inferior vena cava. Surgical resection was performed with the reconstruction of the inferior vena cava using a Dacron prosthesis, the diagnosis of vessel wall leiomyosarcoma was revealed by histopathology. Surgical resection with clear margins remains the only treatment offering the best survival rate. The complex nature of the surgery of those tumors is a major therapeutic challenge for surgeons.

## Introduction

Leiomyosarcoma (LMS) is a rare malignant tumor of the large vessel wall that arises from smooth muscle cells and can develop intraluminal and extraluminal involving adjacent structures. LMS of the inferior vena cava (IVC) is the most common one [1]. This tumor is often diagnosed during the fifth and sixth decades and affects women four times more than men [2]. The IVC LMS diagnosis can be delayed owing to the slow growth and the location in the retroperitoneum. Variable clinical presentation can be seen, ranging from asymptomatic to non-specific symptoms due to compression [3]. The surgical tumor resection with clear margins remains the only treatment improving survival [4].

Through this document, we report the case of a patient with an IVC LMS, the presentation of this tumor, and the surgical treatment modalities.

## Case presentation

**Figure 1:**
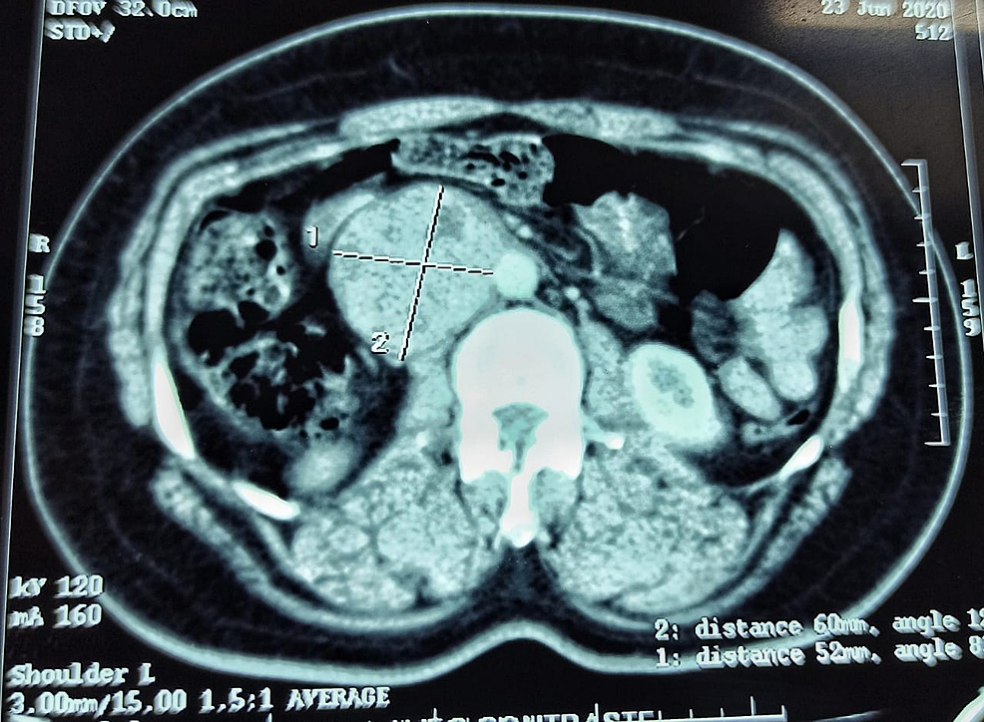
Axial CT of the abdomen reveals a retroperitoneal mass enclosing the inferior vena cava.

A 39-year-old female without a past medical history was referred to our department to treat a right para-aortic retroperitoneal tumor, revealed by pain in the right lumbar fossa with nausea and significant weight loss for six months. No abnormalities were apparent in the physical examination. Laboratory examinations including blood cell counts and renal function tests showed normal values. Computed tomography (CT) revealed a heterogeneous retroperitoneal mass well-defined with a necrotic area, measuring 60 x 57 x 54 mm. This tumor had intimate contact with the aorta and right renal vein with a mass effect on the IVC, which had increased in size. No evidence of metastatic disease was found (Figure 1).

**Figure 2:**
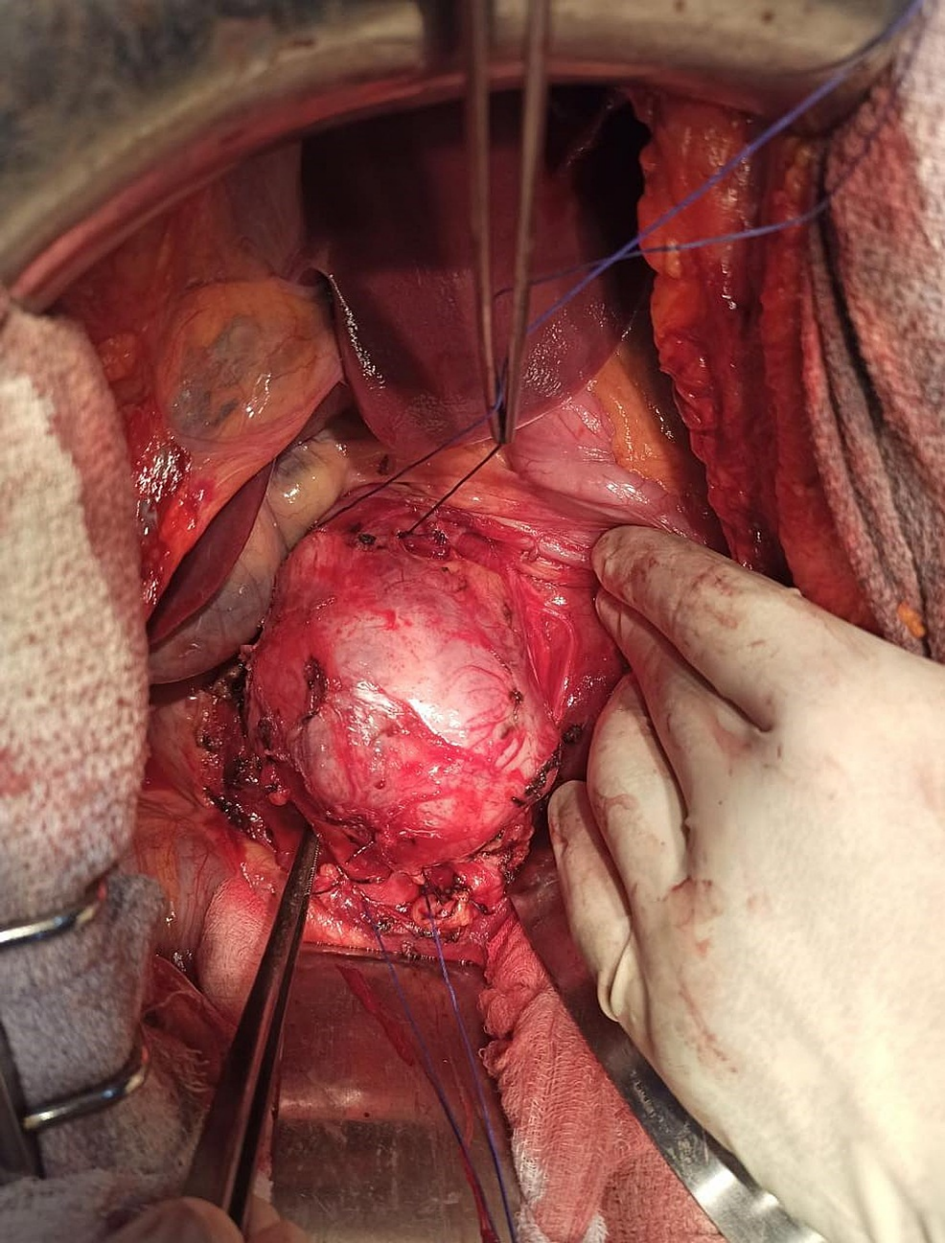
Intraoperative photograph showing the tumor arising from the vena cava.

**Figure 3:**
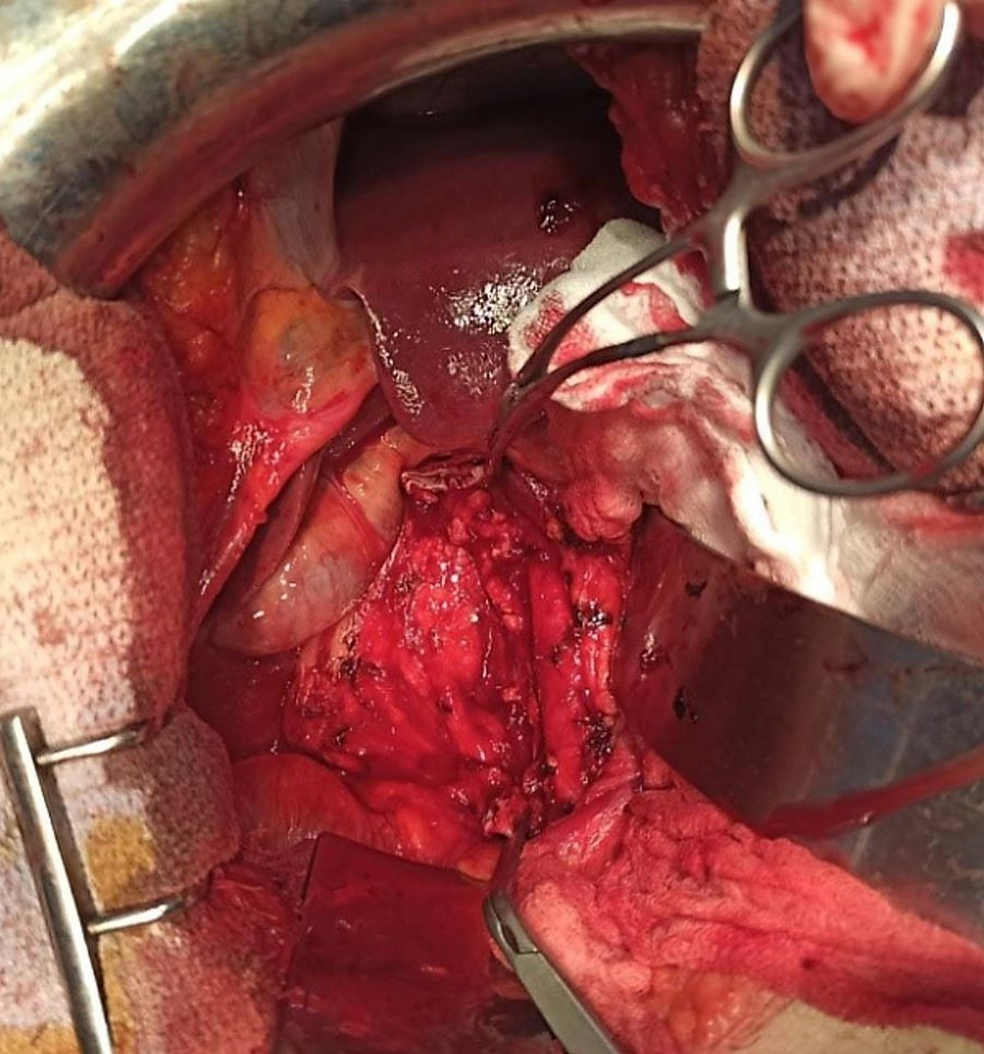
Image showing the infrarenal vena cava clamped after the tumor was removed.

A midline laparotomy was performed, a tumor originating from the wall of the infrarenal IVC was apparent in close contact with the aorta without invading it (Figure 2). After a vascular control with clamps on each side of the tumor, this one was removed (Figure 3).

**Figure 4:**
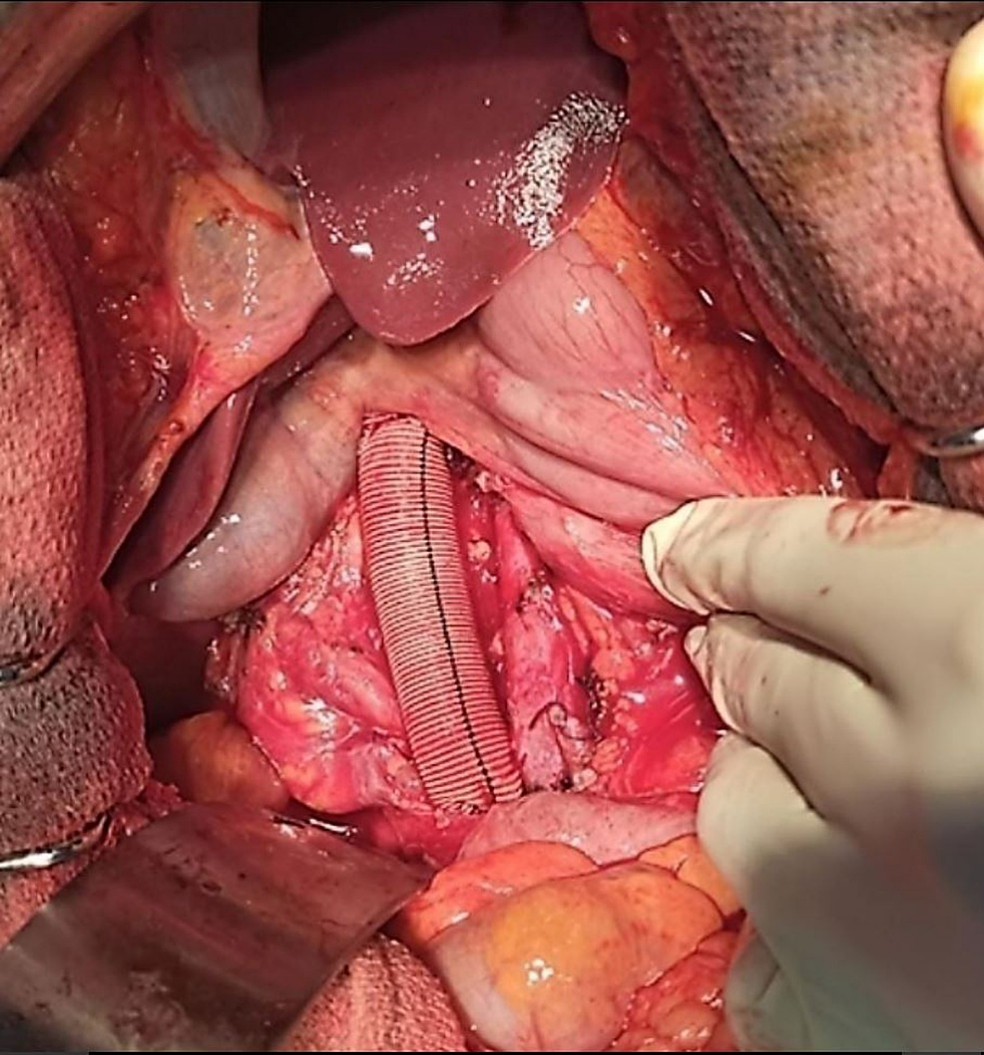
Vascular reconstruction using Dacron graft.

The IVC was reconstructed with Dacron prosthesis implantation using 5-0 Prolene thread, and hemostasis was obtained after removing the clamps (Figure 4). An arteriovenous fistula was performed between the great saphenous vein and the superficial femoral artery. Anatomopathology and immunohistochemistry confirmed the LMS of the IVC with clear surgical margins.

Post-surgery, the patient received a low dose of heparin. On the fifth postoperative day, she developed an intraabdominal hematoma evidenced by acute anemia, that required reintervention for drainage and hemostasis. A CT angiogram was performed a month after surgery showing patency of the prosthesis.

## Discussion

The IVC LMS is a rare malignant tumor that arises from smooth muscle of media. Its metastases are relatively uncommon, and they may be developed in the liver, lungs, lymph nodes, and bones [1,5]. Since the first description of IVC LMS in 1871 by Perl, only 470 cases have been reported in the literature, the majority of them were discovered between the ages of fifty and sixty, with a female predominance [1].

According to their position in the IVC, LMS of vena cava are classified into three types: type I tumor arises from below the renal veins (36% of cases), which was the case of our patient, type II tumor arises from the segment between the renal veins and the hepatic veins (44% of cases), and type III tumors develop from the vena cava above the hepatic veins (20% of cases) [6,7].

The IVC LMS diagnosis may be delayed to late-stage due to its slow progression within IVC, which develops in 5% intraluminally, in 62% extraluminally, and in 33% of cases both intraluminally and extraluminally [8]. The symptoms of LMS depend on the level of involvement of the IVC. IVC LMS type 1 may present with pain in the lower right quadrant, back, flank, and sometimes lower limbs edema. Type 2 may present with epigastric discomfort, while type 3 can cause occlusion of the hepatic veins due to the tumor itself or by the thrombosis, consequently, Budd-Chiari syndrome can appear [9]. The tumor may occur in more than one segment and give several signs and symptoms [10].

CT and MRI scans have a crucial role in the precise diagnosis of LMS by providing information about the tumor's origin and its connection to adjacent structures. These tumors appear as heterogeneous masses with peripheral enhancement and may exhibit areas of hemorrhage and necrosis [11]. Computed tomography venography and contrast magnetic resonance venography provide additional information about the tumor location, its invasion, and a full longitudinal view of the mass [12].

Radical surgical resection removing the entire tumor is the only treatment offering long-term survival [11]. The excision of IVC LMS should be performed in specialized centers by an experienced surgeon to avoid venous injury and major intraoperative bleeding. The tumor should be considered unresectable if the superior mesenteric vessels are involved, and the resection is limited in the case of the suprahepatic vein involvement. However, hepatectomy associated with resection of the IVC and anastomosis of the healthy hepatic vein to a graft has been performed for tumors limited to one hepatic vein or a suprahepatic portion of the inferior vena cava [13,14].

Depending on the level of the IVC involved, the tumor's extension, and the presence or absence of collateral veins, different surgical resection techniques and approaches have been described [15,16]. Before IVC dissection or manipulation, it must be fixed above and below the tumor. Simple clamping is sufficient for infrahepatic IVC reconstruction. For type I, clamping may be achieved between the iliac bifurcation or upper and the suprarenal vena cava. Arterial hypotension and proximal venous hypertension are rare complications and may be treated by the clamp of the infrarenal aorta or the supra celiac aorta [13,17].

After a partial IVC resection, the treatment using a prosthetic patch or direct suture is rarely sufficient. Thus, complete resection is indicated. IVC reconstruction is not always indicated. It depends on the presence of a good network of collaterals, ensuring venous return [16,18]. Several techniques have been described for the IVC reconstruction, and various materials have been used, like synthetic materials including Dacron, polytetrafluoroethylene, vascular graft, or banked vena cava homograft tubularized bovine pericardium, and a stapled peritoneal fascial graft [7]. A small caliber of polytetrafluoroethylene or Dacron is recommended to increase flow velocity and reduce the thrombotic risk [4]. Likewise, the realization of inguinal arteriovenous fistula has been recommended by certain authors to enhance the graft's permeability [1]. Owing to our patient's tumor location and the absence of collateral veins, we opted for the reconstruction of the IVC and the creation of an arteriovenous fistula.

Tumor resection with free margins is the only proven treatment for IVC LMS. Adjuvant radiotherapy or chemotherapy remains a subject of debate [3,19]. Adjuvant treatment was not used in our case. In a study conducted by Wachtel et al., regrouping 377 patients with IVC LMS, the median disease-free survival at one and five years was 57% and 6%, respectively. Furthermore, overall survival was 92% and 55%, respectively. Various prognostic factors for disease-free and overall survival have been found. Isolated involvement of the IVC middle segment is linked to higher disease-free survival. Whereas tumors with a large size (≥9 cm) and adjuvant chemotherapy were associated with decreased disease-free survival. These associations, as well as age greater than 55 years and resection with positive margins, are also prognostic factors reducing overall survival [19].

## Conclusions

IVC LMS is a rare malignant tumor that affects women more than men. The surgical resection with free margins is the unique curative treatment offering the best survival rate. Adjuvant chemotherapy and radiotherapy remain controversial and unclear. The performance of this complex surgery requires a multidisciplinary team of surgeons to have the best results.
